# Highly Informative Ancient DNA ‘Snippets’ for New Zealand Moa

**DOI:** 10.1371/journal.pone.0050732

**Published:** 2013-01-16

**Authors:** Jonathan McCallum, Samantha Hall, Iman Lissone, Jennifer Anderson, Leon Huynen, David M. Lambert

**Affiliations:** 1 Griffith School of Environment and School of Biomolecular and Physical Sciences, Griffith University, Nathan, Queensland, Australia; 2 Institute of Natural Resources, Massey University, North Shore City, New Zealand; University of Otago, New Zealand

## Abstract

**Background:**

Analysis of ancient DNA has provided invaluable information on past ecologies, ancient populations, and extinct species. We used a short snippet of highly variable mitochondrial control region sequence from New Zealand’s moa to characterise a large number of bones previously intractable to DNA analysis as well as bone fragments from swamps to gain information about the haplotype diversity and phylogeography that existed in five moa species.

**Methodology/Principal Findings:**

By targeting such ‘snippets’, we show that moa populations differed substantially in geographic structure that is likely to be related to population mobility and history. We show that populations of *Pachyornis geranoides, Dinornis novaezealandiae,* and *Dinornis robustus* were highly structured and some appear to have occupied the same geographic location for hundreds of thousands of years. In contrast, populations of the moa *Anomalopteryx didiformis* and *Euryapteryx curtus* were widespread, with specific populations of the latter occupying both the North and South Islands of New Zealand. We further show that for a specific area, in this case a North Island swamp, complete haplotype diversity and even sex can be recovered from collections of small, often discarded, bone fragments.

**Conclusions/Significance:**

Short highly variable mitochondrial ‘snippets’ allow successful typing of environmentally damaged and fragmented skeletal material, and can provide useful information about ancient population diversity and structure without the need to sample valuable, whole bones often held by museums.

## Introduction

New Zealand’s flightless moa ratite (Order: Struthioniformes) represented one of the most extensive animal radiations known with at least 9 species in 6 genera [1]. Moa differed significantly in size and shape with each species having a defined paleoecological history. *Dinornis* were the giants of this ratite family, exceeding 200 kg in weight and 200 cm in height, while the coastal moa *Euryapteryx curtus* was only the size of a large turkey and weighed little more than 9 kg [2].

Of all the ancient animals, moa are one of the most studied, with successful DNA extractions from moa bones, coprolites, eggs, and feathers. To date, genetic data from over 300 moa specimens are available, with a number of phylogenies having been constructed in attempts to clarify the number of moa species that existed [1], [3].

Most DNA analyses of moa, and animals in general, have been restricted to selected regions of the mitochondrial genome, with the most studied area being the highly mutagenic hyper-variable-region I (HVRI). The variation inherent in this ∼300 bp region is capable of discriminating not only all moa species, but also harbours a strong phylogeographic signal. Using this region, a large number of moa samples have been successfully genotyped to give a comprehensive understanding of the various moa mitochondrial haplotypes that existed in New Zealand, most during the Holocene period. However, a significant number of gaps in moa phylogeography remain due to difficulty in the amplification of DNA extracted from rare older and/or more degraded bones. A number of these samples derive from regions in New Zealand where moa have been uncharacterised.

DNA damage is driven by moisture, light, and temperature [4] with the best-preserved samples usually being derived from cold, dark, and dry environments such as caves [5]. DNA degradation occurs rapidly post-mortem due to nuclease activity, and is followed more slowly by base modification and further strand breakage due to oxidative and hydrolytic reactions [5]–[10]. As a result, typical amplification limits for any DNAs isolated from skeletal material are approximately 250 bp for mitochondrial DNA and 120 bp for nuclear DNA [3], [11]. Relatively recent studies have shown that amplification success increases substantially if small (<100 bp) DNA fragments are targeted [12], [13], [14].

Use of relatively short highly informative DNA sequences has proved invaluable for typing a number of species, for example, equids [15]. We have previously identified a 30 bp HVRI region in moa capable of distinguishing all moa species and most moa populations, thereby only requiring the amplification of a relatively small 70 bp mitochondrial product (including primer sequences). Here we use this region to genotype a large number of moa bones from isolated regions in New Zealand, the material from which is very low quality and/or very scarce. In addition, we characterize the haplotypes present in a recently discovered swamp in New Zealand’s North Island using only degraded moa bone fragments. We show that these often unrecognisable fragments are able to provide detailed information about the moa populations that occupied this area during the Holocene.

## Results

**Figure 1:**
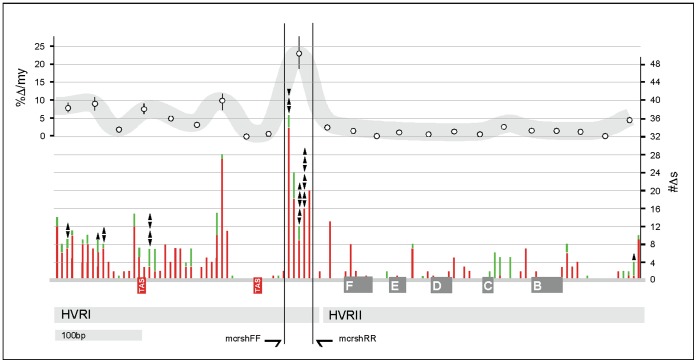
Moa D-loop nucleotide variation. The number of changes was calculated in bins of six nucleotides; shown on the Y axis on the right. Transitions are shown in red, transversions are shown in green. Insertions and deletions are shown by downward and upward pointing arrowheads respectively. Highly-conserved avian F, E, D, C, and E boxes are shown in grey [16]–[18]. The regions covered by the HVRI and HVRII are shown as light grey boxes. TAS sequences are in red. Rates of mutation were calculated in bins of 30 nucleotides and are shown as percentage change per million years (%/Myr) on the left Y axis.

Phylogenetic analyses of approximately 683 bp of the control region of 107 moa representing all known species identified a highly variable 30 bp region capable of not only identifying species, but also populations (Figure S1). This region was estimated to mutate at approximately four times the rate of the surrounding control region at ∼24%/Myr, and is located at the 3′ terminus of the HVRI between a highly conserved F Box and an upstream termination associated sequence (TAS) (Figure 1) [16]–[18]. Secondary structure analysis (using Mfold) of this highly variable region failed to detect conserved structures that may have influenced mutation rate [19]. Conserved primers were designed flanking the 30 bp sequence, to attempt amplification from bones known to be intractable to DNA analysis [3], [11].

**Figure 2:**
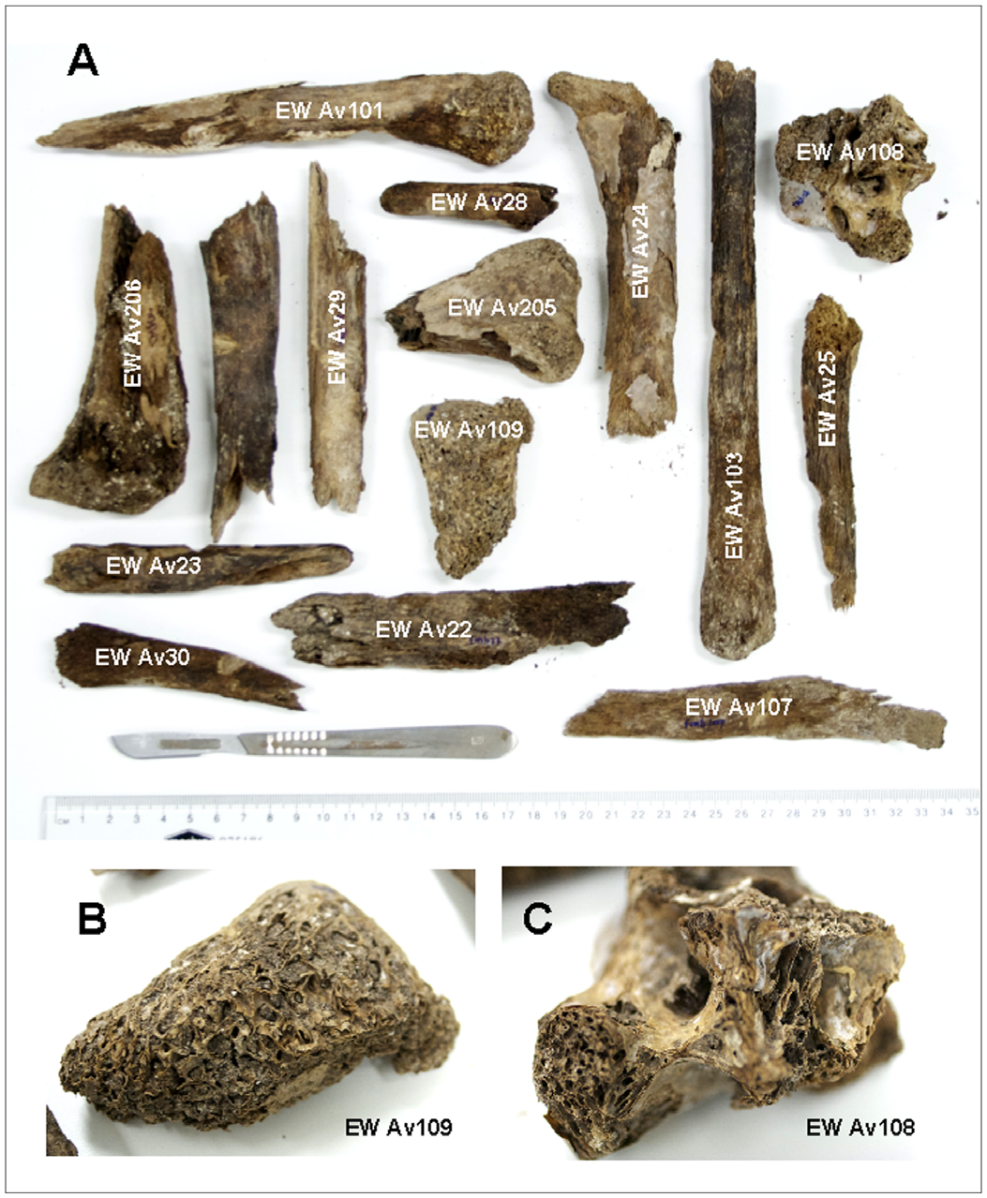
Selected moa bone fragments from Tiniroto swamp. **A**. A selection of degraded moa bone samples recovered from Tiniroto Swamp. **B**. and **C**. Examples of bone fragments successful for aDNA extraction that consisted largely of trabecular material.

Over 40 bones shown previously to be unsuccessful for the amplification of an ∼240 bp PCR product [3], [11] successfully yielded the shorter 30 bp region. These bones included those dated to over 24 kyrBP from Dawson’s Moa Cave in Waitomo, and many from unique, previously unsampled locations within New Zealand (Table 1). Amplification of the shorter 70 bp fragment also proved successful for degraded bone samples recovered from various middens and a swamp recently excavated at Tiniroto on New Zealand’s North Island [20]. Many of these bones exist only as small fragments (see Figure 2) devoid of many recognisable skeletal elements. In some cases they consisted of only light trabecular bone, known to be a difficult source of aDNA (results not shown; Figure 2). Twenty-four moa samples recovered from Tiniroto Swamp were analysed, returning eight haplotypes from three moa species; *Anomalopteryx didiformis*, *Euryapteryx curtus*, and *Dinornis novaezealandiae* (Table 2). All the haplotypes detected by previous whole bone sampling of moa from Tiniroto [20] were recovered by analysis of the shorter fragments. Using newly designed primers moaWF, moaWR, msox9F, and msox9R, the sex of a number of these small bone fragments was determined, and indicated a male:female ratio of 1∶5 at Tiniroto swamp (Table 2).

**Table 1:** Moa samples analysed.

Sample ID	Tissue type	Source	Location	*Species*	*haplotype*	notes
AIM 46/10 E.c?	bone	midden	Taupo	*A. didiformis*	*A1*	Whakamoenga Cave
AIM AR158	bone	midden	Coromandel	*D. novaezealandiae*	*D13*	Onemana
AIM AR2964	bone	midden	Coromandel	*D. novaezealandiae*	*D3*	Hot Water Beach
AIM AR5876	bone	midden	Taupo	*E. curtus*	*E1*	Whakamoenga Cave
AIM t12/500 n53 54/0	bone	midden	Coromandel	*D. novaezealandiae*	*D24*	Wheritoa
AIM t12/26 n49/33	bone	midden	Coromandel	*D. novaezealandiae*	*D23*	Onemana
AIM AR3008	bone	midden	Coromandel	*D. novaezealandiae*	*D24*	Hot Water Beach
AIM AR3292	bone	midden	Coromandel	*E. curtus*	*E14*	Hot Water beach
AIM 2/c16 3/1 n40/6	bone	midden	Coromandel	*E. curtus*	*E1*	Opito
AIM AR5735	bone	midden	Taupo	*D. novaezealandiae*	*D23*	Whakamoenga Cave
AIM 2b18 3/1	bone	midden	Coromandel	*D. novaezealandiae*	*D1*	Opito
AIM B6143	femur	–	Mangaotaki	*P. geranoides*	*P10*	–
AIM B7021	femur	–	Waitanguru	*D. novaezealandiae*	*D10*	–
AIM B7101	femur	–	Doubtless Bay	*D. novaezealandiae*	*D1*	–
AU no lbl. bg G	bone	midden	Houhora	*E. curtus*	*E14*	–
AUG Av5801.7	femur	cave	Waitomo	*A. didiformis*	*A1*	24,800±450 yrBP*
AUG Av5801.8	femur	cave	Waitomo	*A. didiformis*	*A1*	24,800±450 yrBP*
AUG Av5801.9	femur	cave	Waitomo	*E. curtus*	*E1*	24,800±450 yrBP*
CM Av12567	femur	swamp	Te Aute	*D. novaezealandiae*	*D25*	–
CM Av13461	femur	–	Christchurch	*D. robustus*	*Dr11*	–
CM Av15891	femur	–	Kaikoura	*D. robustus*	*Dr4*	–
CM Av16361	femur	–	Castle Hill Basin	*E. curtus*	*E9*	–
CM Av19154	femur	–	Lake Coleridge	*D. robustus*	*Dr16*	–
CM Av19329	femur	swamp	Albury	*D. robustus*	*Dr17*	–
CM Av20607	femur	–	Doubtless Bay	*E. curtus*	*E14*	–
CM Av20621	femur	–	Benmore	*D. robustus*	*Dr8*	Loess ∼10 kyrBP
CM Av20671	tarsometatarsus	midden	Te Rangatupu	*D. novaezealandiae*	*D12*	Site # N129/233
CM Av28271	femur	cave	Inangahua	*D. robustus*	*Dr3*	Limestone cave
CM Av28274	femur	cave	Inangahua	*D. robustus*	*Dr10*	Limestone cave
CM Av36160	femur	swamp	Hamilton’s	*D. robustus*	*Dr13*	–
CM Av9033	femur	swamp	Te Aute	*D. novaezealandiae*	*D3*	–
CM unregistered	femur	reservior	Cashmere	*D. robustus*	*Dr2*	∼8–10 kyrBP
CM SB5	femur	–	Kakanui	*D. robustus*	*Dr18*	
CM SB108	femur	–	Cheviot	*D. robustus*	*Dr1*	juvenile
MNZ S40870.2	phalanx	swamp	Tangatupura	*P. geranoides*	*P6*	18,300±160 yrBP*
OM Av3856	femur	–	Forest Hill	*D. robustus*	*Dr7*	–
OM Av3887	tibiotarsus	–	Karitane	*D. robustus*	*Dr9*	Central Otago
OM Av3935	femur	–	Ngapara	*D. robustus*	*Dr2*	–
OM Av3967	vertebrae	–	Glendhu Bay	*D. robustus*	*Dr13*	Central Otago
DM1	sternum	peat bog	Taihape	*D. novaezealandiae*	*D27*	–
DM2	femur	peat bog	Taihape	*D. novaezealandiae*	*D27*	–
DM3	pelvis	peat bog	Taihape	*D. novaezealandiae*	*D27*	–
DM4	tarsometatarsus	peat bog	Taihape	*D. novaezealandiae*	*D5*	–
DM5	tarsometatarsus	peat bog	Taihape	*D. novaezealandiae*	*D27*	–
DM6	femur	peat bog	Taihape	*E. curtus*	*E14*	–
DM7	tibiotarsus	peat bog	Taihape	*D. novaezealandiae*	*D28*	–
W 1617	femur	swamp	Makirikiri	*E. curtus*	*E2*	ID – T. H. Worthy

In general amplification reactions were carried out once for each sample. To check sequence accuracy, two amplifications were carried out on a subset of samples, and sequenced in both the forward and reverse direction. *radiocarbon date determined from associated bone.

**Table 2:** Moa bone fragments from Tiniroto swamp.

Sample ID	*Species*	*haplotype*	sex
EW Av22	*D. novaezealandiae*	*D3*	F
EW Av23	*D. novaezealandiae*	*D3*	–
EW Av25	*D. novaezealandiae*	*D17*	F
EW Av26	*D. novaezealandiae*	*D17*	–
EW Av27	*D. novaezealandiae*	*D17*	F
EW Av28	*D. novaezealandiae*	*D17*	F
EW Av29	*D. novaezealandiae*	*D3*	M
EW Av30	*A. didiformis*	*A2*	–
EW Av32	*E. curtus*	*E14*	–
EW Av34	*A. didiformis*	*A2*	F
EW Av38	*D. novaezealandiae*	*D17*	–
EW Av100	*D. novaezealandiae*	*D22*	–
EW Av101	*D. novaezealandiae*	*D6*	–
EW Av103	*D. novaezealandiae*	*D5*	F
EW Av104	*A. didiformis*	*A2*	–
EW Av106	*D. novaezealandiae*	*D21*	F
EW Av107	*D. novaezealandiae*	*D21*	–
EW Av108	*D. novaezealandiae*	*D6*	–
EW Av200	*D. novaezealandiae*	*D3*	–
EW Av204	*A. didiformis*	*A2*	–
EW Av205	*D. novaezealandiae*	*D3*	F
EW Av206	*A. didiformis*	*A2*	F
EW Av207	*A. didiformis*	*A2*	F
EW Av332	*D. novaezealandiae*	*D3*	–

Sample haplotype and sex are shown.

All bones sampled represented five of the nine currently recognised moa species, and consisted of *Pachyornis geranoides, A. didiformis, Dinornis robustus, D. novaezealandiae,* and *E. curtus*. Combining our data with that previously published [1], [3], [11], [20]–[23], the total number of haplotypes detected in each species for the highly variable 30 bp region was 14 (*P. geranoides*), 4 (*A. didiformis*), 17 (*D. robustus*), 28 (*D. novaezealandiae*), and 18 (*E. curtus*). As most locations are represented by only one or two samples, an approximate indication of phylogeographic genetic diversity within each of these species can be obtained by simply dividing the number of haplotypes by the number of locations from which samples were sourced. Relatively high ratios, suggesting high phylogeographic/genetic diversity, were obtained for *D. novaezealandiae* (1.1), *P. geranoides* (0.58), *D. robustus* (0.5), and *E. curtus* (0.72) while a very low ratio was obtained for the moa *A. didiformis* (0.13). As most moa bones date to the Holocene [1], [3] (<∼12 kyrBP), species diversity detected is restricted to this time period. The few samples older than Holocene (AUG Av5801.7–9, 24 kyrBP; MNZ S40870.1, 18.3±0.3 kyrBP; Table 1) harboured haplotypes found in Holocene moa from the same approximate location, suggesting that some moa populations have remained in the same general area for thousands of years.

**Figure 3:**
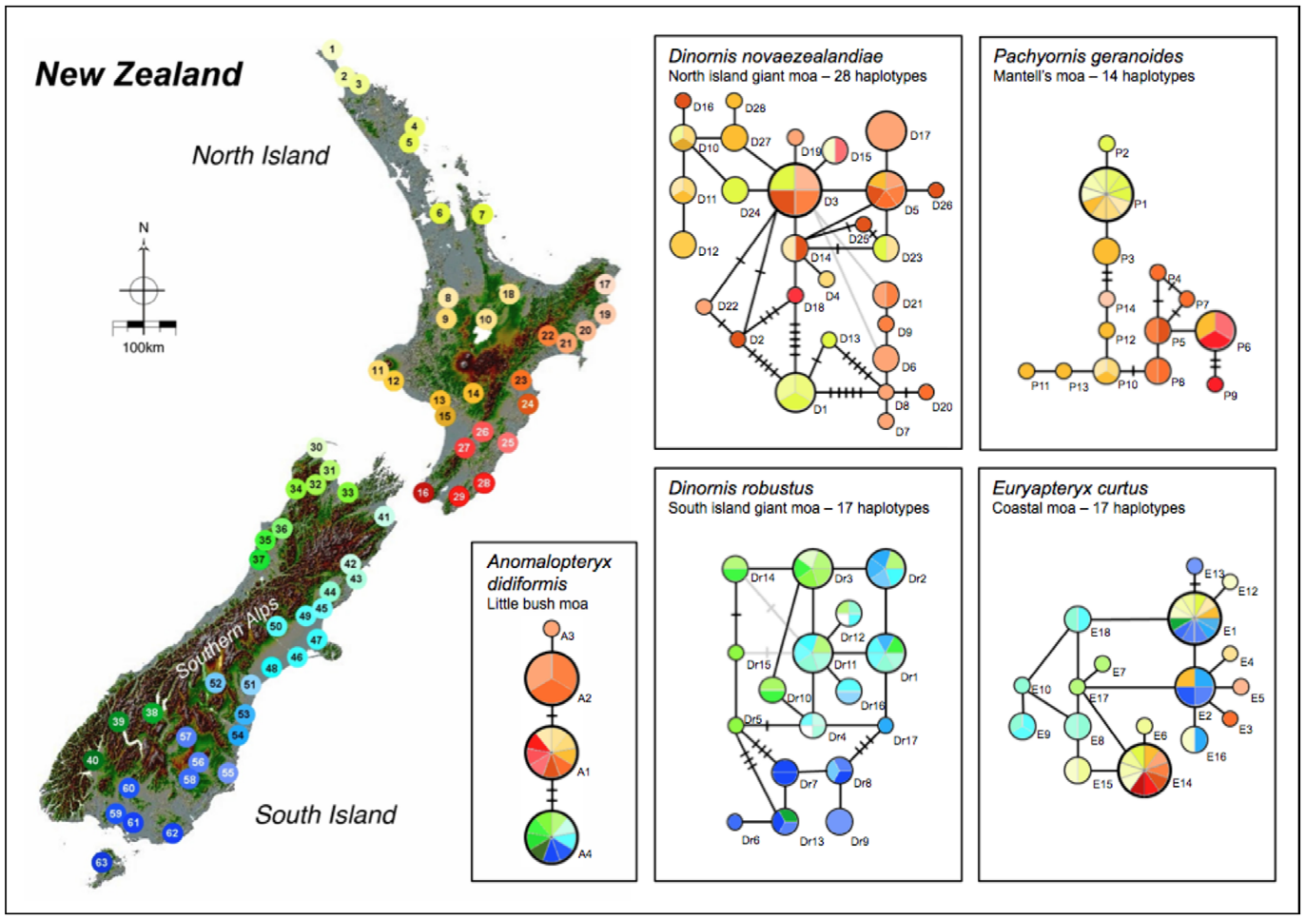
Location and spanning network analysis of moa haplotypes. Dots on the map of New Zealand (left) represent sample locations, identified by a unique color code and number (key in Figure S2). Sequences were aligned in ClustalW [35]. Haplotype networks (right) were constructed using TCS 1.21 [31] (with gaps treated as the fifth state) and are coloured according to sample location. Unknown locations are shown by clear circles. Circles are labeled in the network with haplotype number (eg P10 - *Pachyornis geranoides* haplotype 10). The circle size indicates the frequency of each haplotype; small for 1 occurrence, medium for 2–4, large for 5–10 and the largest circles for more than 10. Lines between haplotypes show single mutation events and linebreaks indicate intermediate, as yet undetected haplotypes. Light grey lines show possible additional linkages.

Spanning network analyses of haplotypes (Figure 3) show that the East Coast of New Zealand’s North Island and the West Coast of the South Island were particularly rich in haplotype diversity (Figure 3). In the North Island, haplotypes for *P. geranoides* and *D. novaezealandiae*, can be divided into predominantly eastern and western variants. In addition, moa bones sampled from middens in the Coromandel area recovered three previously uncharacterized haplotypes for *D. novaezealandiae*: D13, D23, and D24 (Table 1). *D. robustus* haplotypes south of the Hawkdun and Kakanui mountain ranges in the South Island, and *D. novaezealandiae* haplotypes in the far north of the North Island are separated from their nearest neighbours by several mutations. A distinct North Island/South Island separation is evident for haplotypes of *A. didiformis,* while surprisingly, for *E. curtus* three haplotypes, E1, E2, and E16, are found on both islands.

## Discussion

The highly variable HVRI ‘snippet” detailed here has been used to determine genetic variability in five moa species. This region is capable of identifying moa species as well as populations. Similar highly variable regions have been detected at comparable genomic locations in a number of animals including horse, bison, dog [24], chicken, human [25], elephant [26], and snake [27]. Secondary structure analysis of the HVRI region in moa failed to detect conserved structures that may have influenced mutation rates within the ∼30 bp ‘snippet’ (data not shown), suggesting that perhaps DNA-protein complexes formed in the HVR during mitochondrial replication may result in the formation of single-stranded DNAs in the ∼30 bp region that are more prone to mutation.

Using degraded samples previously unsuccessful for the amplification of larger mitochondrial fragments, we were able to recover new informative sequences from over 40 moa bones, from new locations. We obtained new mitochondrial haplotypes for the five species studied, suggesting that population diversity amongst moa is greater than previously thought. The large number of new haplotypes detected suggests that haplotype saturation is unlikely to have been attained, even for the haplotype-poor *A. didiformis*, indicating diverse moa populations during the Holocene.

Although caution must be exercised in making inferences about populations using such a short fragment, we note that the distribution patterns and relationships amongst the haplotypes are very similar to those shown in phylogenetic trees constructed by Bunce et al [1] and Baker et al [3]. We show considerable geographic structure for the two lowland giant moa, *D. novaezealandiae* and *D. robustus*, with most genetic variation detected in the east of the North Island and the west of the South Island respectively. Both these regions are characterized by rich mountainous terrains able to sustain diverse habitats, even during the Pleistocene glacial and interglacial periods, where population movement was likely to be limited [28]. For both *D. novaezealandiae* and *P. geranoides*, a haplotype split is evident separating the east and west coast of the North Island. For *P. geranoides* in particular, the split is demarcated by mountain ranges in the central North Island. The location-specific nature of a number of haplotypes suggests that many populations of *Dinornis* and *P. geranoides* have remained geographically static for possibly hundreds of thousands of years.

The spanning network of *Euryapteryx curtus* sequences (Figure 3) fails to show any obvious geographic pattern, with three haplotypes (E1, E2, and E16) present in both the North and South Island. This pattern has not been recorded for any other moa species. The primarily coastal habitat occupied by *Euryapteryx curtus* was likely to have been subjected to large scale terrain changes during glacial cycles of the Pleistocene, with the subsequent formation of land bridges (∼100 kyrBP and ∼450 kyrBP) joining the North and South Island. These bridges would have inevitably provided opportunities for population mixing [1]. For *A. didiformis*, few haplotypes were found, with a single haplotype covering all samples from the South Island and only three being detected in the North Island. This may be the result of a bottleneck in this species, followed by rapid intra-island expansion.

We also analysed 11 moa samples from middens in the Taupo and Coromandel region in the North Island. Three haplotypes (D13, D23, and D24) from the Coromandel area were not found anywhere else and may be indicative of this general region. Our small sample size and the absence of moa samples from natural sites in these areas did not allow us to determine whether early moa hunters may have transported moa from neighbouring locations [29].

Thirty degraded moa bone fragments were analysed from Tiniroto Swamp, near Gisborne on New Zealand’s North Island. Twenty-four of these bone fragments were succesful for the amplification of the ‘snippets’, with a surprisingly large number (11) also allowing amplification of sex-specific nuclear DNA markers [11]. A total of eight haplotypes were detected from 24 samples, and included all haplotypes detected by previous aDNA analysis of high quality complete bones [20] suggesting that comprehensive datasets can be obtained from ‘leftover’ bone fragments. From the sexing results, as expected, the ratio was heavily skewed in favour of females, as has been shown previously for moa from other swamps [30]. This may be due to behavioural differences between the sexes, where the male is responsible for tending the nest, while the female forages for food [22], [30].

We show here that small DNA fragments recovered from small, highly degraded and/or old bones may provide useful information on species and populations. The ability to characterize animal populations using very short miochondrial ‘snippets’, from often discarded bone samples may in addition prove useful for the study of species where the destructive sampling of valuable whole bones is undesirable, such as those used by museums for display purposes or morphological analysis.

## Materials and Methods

### Samples

Moa bone samples were kindly supplied by the following: Canterbury Museum (CM), Auckland Institute and Museum (AIM), University of Auckland (AU), University of Auckland Geology Dept (AUG), Otago Museum (OM), Whanganui Regional Museum (W), the Museum of New Zealand Tongarewa (MNZ), and the private collection of Derek Mickelson, Taihape (DM).

### Ancient DNA Extraction

All bone samples were treated using criteria set for the analysis of DNA from ancient material [33]. DNA was extracted from the bone by scraping shavings from the bone surface using a scalpel. Bone scrapings were then extracted for DNA following a modified procedure of Huynen et al [11]. The exact method used is outlined below.

Approximately 50 mg of bone scrapings were incubated with rotation overnight in 0.8 ml of 0.5 M EDTA/0.001% Triton X100, then centrifuged, the EDTA/Triton X100 was decanted, and 0.4 ml of SET buffer (50 mM NaCl, 5 mM EDTA, 50 mM Tris-Cl pH 7.6) was added with 40 ul of 10% SDS, approximately 0.5 mg of dry dithiothreitol (DTT), and 0.5 mg of dry proteinase K. The mix was incubated with rotation at 56°C overnight, then extracted with phenol/chloroform followed by chloroform, before 200 ul of the extract was purified using a Qiagen DNeasy® Blood and Tissue Kit. The DNA was finally eluted from the silica column with 40 ul of 0.001% Triton X100.

*Ancient DNA precautions*: All DNA extractions were carried out in a physically separate, dedicated ancient DNA laboratory. This facility is separated by 500 metres from the main laboratory in another building where amplifications were performed. A number of sequences were obtained in both directions from separate amplifications and in some cases from multiple extractions. Sequences from several samples were verified by LH at Massey University’s Ancient DNA facility in Auckland, New Zealand.

### DNA Amplification and Sequencing

Approximately 2 ul of DNA was amplified by polymerase chain reaction (PCR) in 10 ul volumes containing 50 mM Tris-Cl pH 8.8, 20 mM (NH_4_)_2_SO_4_, 2.5 mM MgCl_2_, 1 mg/ml BSA, 200 uM each of dGTP, dUTP, dCTP, and dATP, 0.5 uM of each primer mcrshFF and mcrshRR [22], 0.06 U of cod Uracil-DNA Glycosylase (ArcticZymes), and ∼0.3 U of platinum Taq (Invitrogen). The reaction mix was incubated at room temp for approximately 15 min and then subjected to amplification in an ABI GeneAmp® PCR System 9700 using the following parameters: 94°C for 2 min (×1), 94°C for 20 sec, 58°C for 1 min (×45). Amplified DNAs were detected by agarose gel electrophoresis in 0.5×Tris-borate-EDTA buffer (TBE), stained with 50 ng/ml ethidium bromide in TBE, and then visualized over UV light. Positive amplifications were purified by centrifugation through ∼40 ul of dry Sephacryl™ S200HR and then sequenced using primer F+6t (5′TTTTTTAGTCGACGCTTCTAGCTT) or R (5′CATGCTACCTGCTACTGT) at the Griffith University DNA Sequencing Facility using Applied Biosystems (ABI) BigDye® Terminator v3.1 chemistry and an ABI 3730 Genetic Analyzer.

*Molecular sexing*: DNA extractions from Tiniroto Swamp samples were sexed in three steps. First, all samples were tested by PCR for the presence of a 58 bp W chromosome product using the W-specific primers moaWF (5′CACTGTTTTCTTACTAATAGCGAAGT) and moaWR (5′ATGTTAAGCAATGCTCTATGACA). Second, negative samples were tested for the presence of amplifiable nuclear DNA by targeting a 72 bp autosomal PCR product using the moa-specific autosomal primers msox9F (5′CTGCTCCTTGAATCTGATGA) and msox9R (5′CTTAGGCCAACGATACGAAA). Third, samples that amplified using the autosomal primers, were retested using the W-specific primers.

### Bioinformatics

Phylogenetic rates (Figure 1) for ∼683 bp of the 5′ terminus of the moa control region were calculated in PAUP*4.0 b. The 5′ control region sequences of 107 moa samples covering all known species were initially aligned in Sequencher® (Gene Codes) and then realigned by eye, with minimal gap insertion. Maximum likelihood trees were constructed in PAUP*4.0 b and each of the ∼683 bases was analysed to determine base change type (transition, transversion, insertion, or deletion) and mutation rate. Moa control region mutation rates were obtained from Bunce et al [1].

Minimum spanning networks were constructed for each species using a highly variable control region segment from bases 554–580 (amplified by mcrshFF and mcrshRR; numbering from the complete mitochondrial genome of *D. robustus*; NC_002672) in TCS 1.21 [31].

DNA folding was determined using the programme Mfold [17] with the free energy rules of SantaLucia, Jr [32]. [NaCl] and [MgCl_2_] were adjusted in the programme to approximate physiological concentrations of 150 mM and 0.2 mM respectively [34].

## Supporting Information

Figure S1 **Phylogenetic analysis of 107 moa.** Approximately 683 bp of moa control region sequence was aligned by eye and a distance neighbour-joining phylogenetic tree was constructed in PAUP*4.0b. 1000 bootstrap replicates were carried out and all major nodes have >60% support. *Pg - Pachyornis geranoides, Pe - Pachyornis elephantopus, Pa - Pachyornis australis, Ecu - Euryapteryx curtus, Ad - Anomalopteryx didiformis, Ec - Emeus crassus, Dn - Dinornis novaezealandiae, Dr - Dinornis robustus, Md - Megalapteryx didinus.*
(TIFF)

Figure S2 **Sample locations.** The location of samples analysed is shown. Locations separated by less than ∼25 km are grouped as a single location.
(TIFF)
