# Supplementary figures and images for: Highly Informative Ancient DNA ‘Snippets’ for New Zealand Moa
Source: PLoS One. 2013 Jan 16;8(1):e50732. doi: 10.1371/journal.pone.0050732 (PMC3547012; doi:10.1371/journal.pone.0050732)

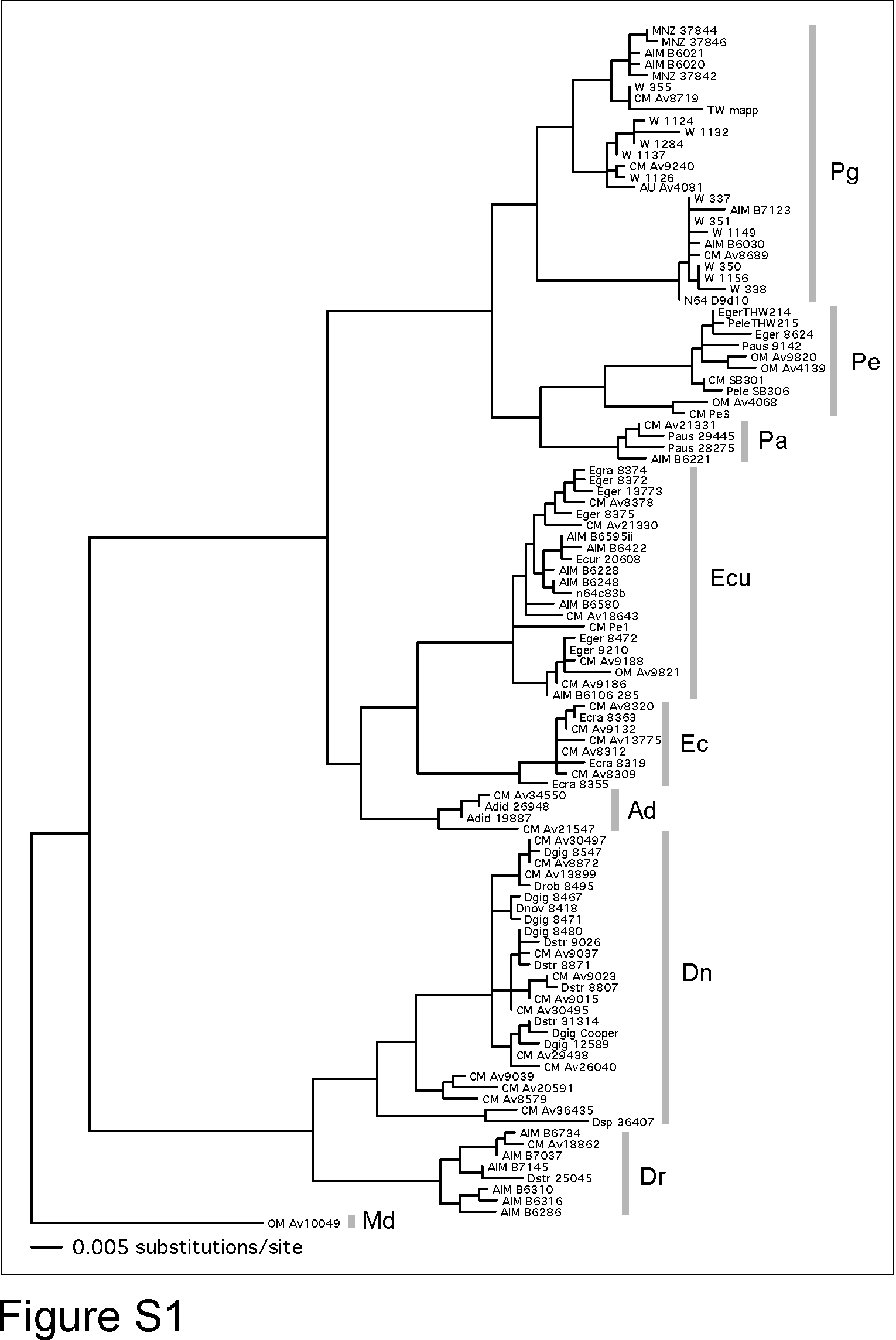

Supplement: Figure S1 — Phylogenetic analysis of 107 moa. Approximately 683 bp of moa control region sequence was aligned by eye and a distance neighbour-joining phylogenetic tree was constructed in PAUP*4.0b. 1000 bootstrap replicates were carried out and all major nodes have >60% support. Pg - Pachyornis geranoides, Pe - Pachyornis elephantopus, Pa - Pachyornis australis, Ecu - Euryapteryx curtus, Ad - Anomalopteryx didiformis, Ec - Emeus crassus, Dn - Dinornis novaezealandiae, Dr - Dinornis robustus, Md - Megalapteryx didinus. (TIFF) [file pone.0050732.s001.tiff]

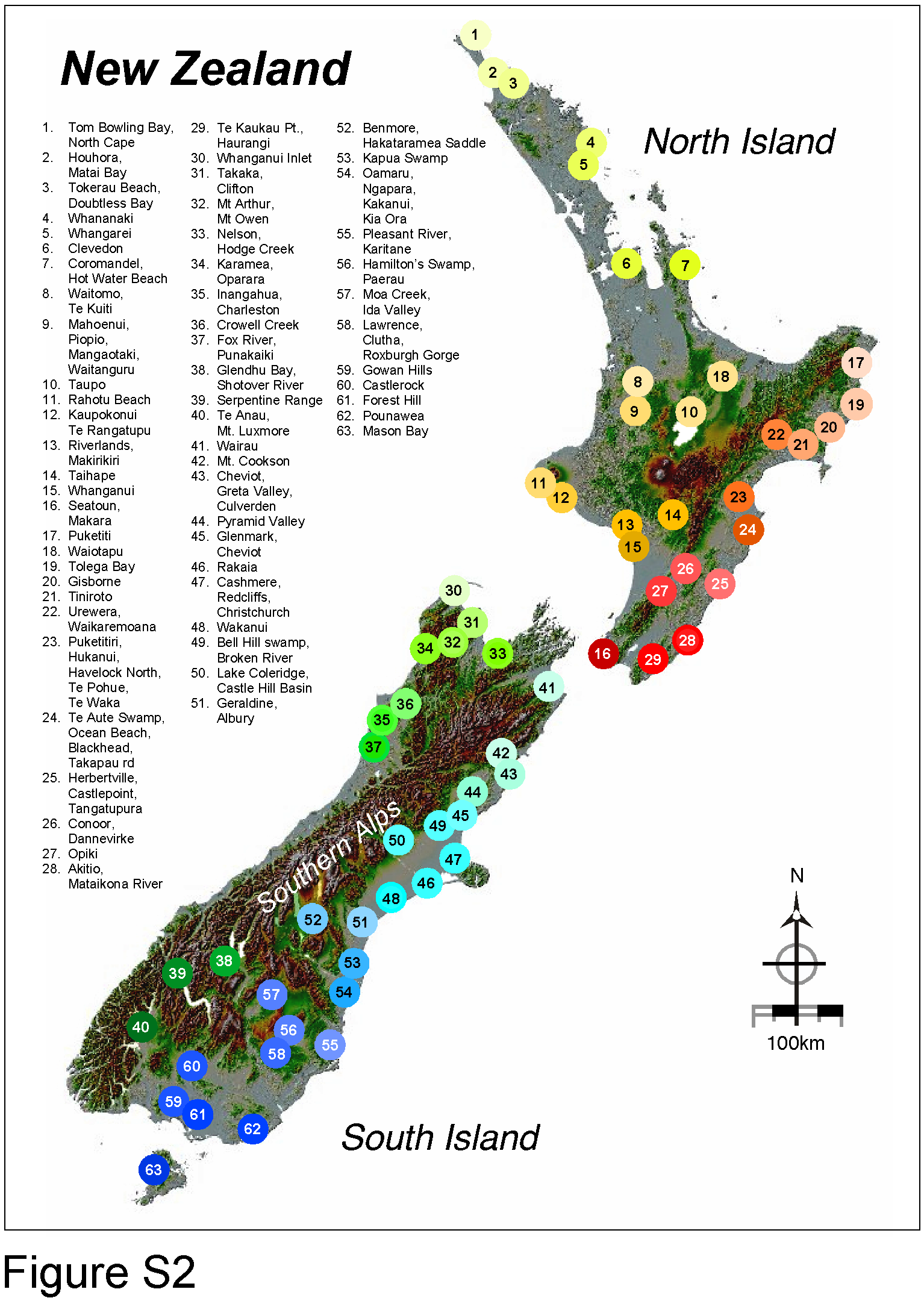

Supplement: Figure S2 — Sample locations. The location of samples analysed is shown. Locations separated by less than ∼25 km are grouped as a single location. (TIFF) [file pone.0050732.s002.tiff]
